# Upregulation of *REG IV* gene in human intestinal epithelial cells by lipopolysaccharide via downregulation of microRNA‐24

**DOI:** 10.1111/jcmm.17498

**Published:** 2022-08-09

**Authors:** Shin Takasawa, Chikatsugu Tsuchida, Sumiyo Sakuramoto‐Tsuchida, Tomoko Uchiyama, Mai Makino, Akiyo Yamauchi, Asako Itaya‐Hironaka

**Affiliations:** ^1^ Department of Biochemistry Nara Medical University Kashihara Japan; ^2^ Department of Diagnostic Pathology Nara Medical University Kashihara Japan

**Keywords:** inflammatory bowel diseases, LPS, microRNA‐24, *REG IV*

## Abstract

The pathophysiology of inflammatory bowel diseases (IBD) reflects a balance between mucosal injury and reparative mechanisms. Some regenerating gene (*Reg*) family members (*REG Iα*, *REG Iβ* and *REG IV*) are expressed in Crohn's disease (CD) and ulcerative colitis (UC) and involved as proliferative mucosal factors in IBD. We revealed that *REG Iα* and *REG Iβ* were induced in cell culture system by IL‐6/IL‐22. Although *REG IV* was upregulated in IBD biopsy samples, the upregulation of *REG IV* was not at all induced in cell culture by autoimmune‐related cytokines such as IL‐6, IL‐22 and TNFα. Here, we analysed *REG IV* expression in LS‐174 T and HT‐29 human intestinal epithelial cells by real‐time RT–PCR and elisa. REG IV expression was induced by lipopolysaccharide (LPS). However, LPS did not activate *REG IV* promoter activity. As the LPS‐induced upregulation of *REG IV* was considered to be regulated post‐transcriptionally, we searched targeted microRNA (miR), which revealed that *REG IV* mRNA has a potential target sequence for miR‐24. We measured the miR‐24 level of LPS‐treated cells and found that the level was significantly lower. The LPS‐induced increase of *REG IV* mRNA was abolished by the introduction of miR‐24 mimic but not by non‐specific control RNA.

## 1 INTRODUCTION

Crohn's disease (CD) and ulcerative colitis (UC), the two primary forms of idiopathic human inflammatory bowel disease (IBD), are both characterized by chronic, destructive intestinal inflammation of unknown cause(s). Despite advances over the past decade in our understanding of the cellular and molecular mechanisms underlying chronic inflammation, the precise aetiopathogenic factors in IBD remain undefined.

Regenerating gene (*REG*) family proteins are structurally similar proteins belonging to the calcium‐dependent (C‐type) lectin superfamily.^1^ In humans, five *REG* family genes (i.e. *REG Iα*, *REG Iβ*, *REG*‐related sequence [*RS*] [pseudogene], hepatocarcinoma‐intestine‐pancreas/pancreatitis‐associated protein [*HIP/PAP*] [islet neogenesis associated protein: *INGAP*] and *REG III*) are tandemly ordered on chromosome 2p12, while *REG IV* is located on chromosome 1q12‐q21.^1^ The first *Reg* was discovered in regenerating pancreatic islets from 90% pancreatectomized rats receiving poly(ADP‐ribose) polymerase (PARP) inhibitor, nicotinamide.^2^, ^3^ Reg proteins have since been found in other physiological and pathophysiological processes. Their basic biological effects seem to be induction of cellular proliferation.^1^, ^4^ Reg family proteins have been also suggested to be involved in cellular proliferation in gastrointestinal cells.^5^, ^6^ Elsewhere in the gastrointestinal system, the proteins are found during tissue injury.^1^ They are also overexpressed in gastric and colorectal cancers and in colorectal cancer cell lines.^1^, ^7^, ^8^

Concerning IBD, overexpression of *REG Iα* and *REG Iβ* mRNA in resected colonic tissue from CD and UC was reported.^9^ We also showed the overexpression of *HIP/PAP* and *REG III* in IBD.^10^ Overexpression of *REG Iα* mRNA and protein in UC, particularly in dysplasia or cancer, and a possible role for REG Iα as a marker of UC‐associated neoplasia were also reported. Recently, van Beelen Granlund et al. analysed four *REG* family genes (*REG Iα*, *REG Iβ*, *HIP/PAP* and *REG IV*) in five functional human *REG* family members and found the genes were overexpressed in IBD samples.^11^

We recently examined all five *REG* family genes in IBD and found overexpression of *REG Iα*, *REG Iβ*, and *REG IV* mRNAs in CD and of *REG IV* mRNA in UC. Reporter gene assays and small inhibitory RNA (siRNA)‐mediated knockdown experiments indicated that the overexpression of *REG Iα* and *REG Iβ* mRNA was mediated through several transcription factors, including myeloid zinc finger 1, related transcriptional enhancer factor‐1/TEA domain transcription factor 4, and signal transducer and activator of transcription 3 in *REG Iα*; helicase‐like transcription factor/forkhead box protein N2 in *REG Iβ*.^12^, ^13^ However, induction mechanism of REG IV in IBD has been unclear. In the present study, we revealed REG IV was induced by lipopolysaccharide (LPS) and that the induction was abolished by the introduction of siRNA for LPS receptors (receptor for advanced glycation endproducts [RAGE] and Toll‐like receptor [TLR] 4) and microRNA (miR)‐24 mimic.

## 2 MATERIALS AND METHODS

### 2.1 Cell culture

LS‐174 T and HT‐29 human intestinal epithelial cells were grown in RPMI 1640 (Nacalai Tesque) supplemented with 10% foetal bovine serum (Sigma), 100 U/ml penicillin and 100 μg/ml streptomycin (FUJIFILM Wako Pure Chemical Co.) as described in previous literature.^12^, ^13^ LPS was purchased from FUJIFLM Wako. Advanced glycation endproducts (AGE)‐bovine serum albumin (BSA) was purchased from Calbiochem®, Merck KGaA, high mobility group box (HMGB)1 was from Bio‐Techne, and S100 Ca^2+^‐binding protein B (S100B) was from Medical & Biological Laboratories Co., Ltd. Cells were treated with 1 μg/ml LPS, 150 μg/ml AGE‐BSA, 1 μg/ml HMGB1 or 100 ng/ml S100B for 24 h, as described in the literature.^14^, ^15^

### 2.2 Measurement of viable cell numbers by tetrazolium salt cleavage

LS‐174 T and HT‐29 cells (2 × 10^4^ cells/100 μl in 96‐well plate) were incubated at 37°C overnight, and the siRNA against REG IV and scrambled RNA (control) were introduced and incubated at 37°C for 24 h in the presence of 0–30 μg/ml LPS. The viable cell numbers were determined by a Cell Counting kit‐8 (Dojindo Laboratories) according to the manufacturer's instructions. Briefly, WST‐8 (2‐(2‐methoxy‐4‐nitrophenyl)‐3‐(4‐nitrophenyl)‐5‐(2,4‐disulfophenyl)‐2H‐tetrazolium monosodium salt) solution was added to cells in 96‐well plates, and the cells were incubated at 37°C for 30 min. The optical density of each well was read at 450 nm (reference wave length at 650 nm) using a Sunrise™ microplate reader (Tecan), as described.^14^, ^16^, ^17^, ^18^

### 2.3 Real‐time reverse transcriptase‐polymerase chain reaction (RT–PCR)

Total RNA was isolated using a RNeasy Protect Cell Mini kit (Qiagen) from LS‐174 T and HT‐29 human intestinal epithelial cells, and cDNA was synthesized from total RNA as a template using a High Capacity cDNA Reverse Transcription kit (Applied Biosystems) as described in previous studies.^12^, ^13^, ^14^, ^15^, ^16^, ^17^, ^18^, ^19^ The cDNA was subjected to PCR with the following primers: *β‐actin* (NM_001101) sense primer, 5’–GCGAGAAGATGACCCAGA–3′ and antisense primer, 5′–CAGAGGCGTACAGGGATA–3′; *REG Iα* (NM_002909) sense primer, 5’–AGGAGAGTGGCACTGATGACTT–3′ and antisense primer 5’–TAGGAGACCAGGGACCCACTG–3′; *REG Iβ* (NM_006507) sense primer, 5’–GCTGATCTCCTCCCTGATGTTC–3′ and antisense primer, 5′–GGCAGCTGATTCGGGGATTA–3′; *REG III* (AB161037) sense primer, 5’–GAATATTCTCCCCAAACTG–3′ and antisense primer, 5’–GAGAAAAGCCTGAAATGAAG–3′; *HIP/PAP* (NM_138937) sense primer, 5’–AGAGAATATTCGCTTAATTCC–3′ and antisense primer, 5’–AATGAAGAGACTGAAATGACA–3′; *REG IV* (AY007243) sense primer, 5’–ATCCTGGTCTGGCAAGTC–3′ and antisense primer, 5’–CGTTGCTGCTCCAAGTTA–3′, and *RAGE* (NM_001136) sense primer, 5’–TGGAACCGTAACCCTGACCT–3′ and antisense primer, 5’–CGATGATGCTGATGCTGACA–3′. All the PCR primers were synthesized by Nihon Gene Research Laboratories (NGRL). Real‐time PCR was performed using KAPA SYBR® FAST qPCR Master Mix (Kapa Biosystems) and Thermal Cycler Dice Real Time System (Takara Bio Inc.) as described in the literature.^12^, ^13^, ^14^, ^15^, ^16^, ^17^, ^18^ PCR was performed with an initial step of 3 min at 95°C followed by 40 cycles of 3 s at 95°C and 20 s at 60°C for *β‐actin*, *REG III*, *HIP/PAP* and *RAGE*, and 40 cycles of 3 s at 95°C and 20 s at 64°C for *REG Iα, REG Iβ* and *REG IV*. Target cDNAs were cloned into pBluescript SK(−) plasmid (Stratagene), and sequential 10‐fold dilutions from 10^2^ ~ 10^7^ copies/μl were prepared. The serial dilutions were run to verify the specificity and to test the sensitivity of the SYBR Green‐based real‐time RT‐PCR. Target mRNA value was normalized to that of *β‐actin* mRNA, which was used to account for differences in the efficiency of reverse transcription between samples.

### 2.4 Measurement of REG IV in culture medium by enzyme‐linked immunosorbent assay (elisa)

Human LS‐174 T and HT‐29 intestinal epithelial cells were exposed to 1 μg/ml LPS for 24 h, culture medium was collected, and the concentration of REG IV was measured by using Human REG IV elisa kit (Cloud‐Clone) according to the instructions of the supplier.

### 2.5 Construction of reporter plasmid and luciferase assay

Reporter plasmid was prepared by inserting the promoter fragments of human *REG IV* (−1053 ~ +22) upstream of a firefly luciferase reporter gene in the pGL3‐Basic vector (Promega). The reporter plasmid was transfected into human LS‐174 T and HT‐29 intestinal epithelial cells using Lipofectamine® 3000 (Invitrogen), as described in the literature,^12^, ^13^ and the cells were stimulated by LPS (1 μg/ml) for 24 h. The cells were harvested, and cell extracts were prepared in extraction buffer (0.1 M potassium phosphate, pH 7.8/0.2% Triton X‐100; Life Technologies). To monitor transfection efficiency, pCMV•SPORT‐βgal plasmid (Life Technologies) was co‐transfected in all experiments at a 1:10 dilution. Luciferase activity was measured using a PicaGene luciferase assay system (Toyo‐ink) and was normalized by the β‐galactosidase activity^12^, ^13^, ^14^, ^17^, ^18^, ^19^ using Beta‐Glo® Assay System (Promega).

### 2.6 MiRNA extraction, reverse transcription and real‐time quantitative PCR

Total RNA including microRNA (miRNA) was isolated from LS‐174 T and HT‐29 intestinal epithelial cells using the miRNeasy Mini Kit (Qiagen) according to the manufacturer's instructions. An equal amount of DNase‐treated RNA was Poly‐A tailed using a Mir‐X™ miRNA first strand synthesis kit (Clontech Laboratories, Inc.) according to the manufacturer's protocol. The condition for PCR was 95°C for 10 s, followed by 45 cycles of amplification (95°C, 5 s, 60°C, 20 s). U6 small nuclear RNA was used as an endogenous control for miRNA as described in previous studies.^13^, ^17^ The cDNA was subjected to PCR with the following primers: miR‐24 sense primer, 5’–TGGCTCAGTTCAGCAGGAACA–3′ and antisense primer, 5’–GTGCAGGGTCCGAGGT–3′; U6 sense primer, 5’–CTCGCTTCGGCAGCACA–3′ and antisense primer, 5’–AACGCTTCACGAATTTGCGT–3′.

### 2.7 MiR‐24 mimic transfection

MiR‐24 mimic (5′–UGGCUCAGUUCAGCAGGAACAGtt–3′, 5′–CUGUUCCUGCUGAACUGAGCCAtt–3′) and non‐specific control RNA (miR‐24 mimic NC) (5′–UUCUCCGAACGUGUCACGUtt–3′, 5′–ACGUGACACGUUCGGAGAAtt–3′) were synthesized by NGRL and introduced into human LS‐174 T and HT‐29 intestinal epithelial cells using Lipofectamine® RNAiMAX (Invitrogen)^19^, ^26^ just before LPS stimulation, and the mRNA levels of *REG IV* were measured by real‐time RT‐PCR, as described in previous studies.^12^, ^13^, ^14^, ^17^

### 2.8 RNA interference (RNAi)

Small interfering RNAs (siRNAs) directed against human REG IV, RAGE and TLR4 mRNAs were synthesized by NGRL. The sense sequences of siRNA for human REG IV, *RAGE* and *TLR4* were 5’–CAUGCUUCUGGAAGCCAUCtt–3′, 5′–AUCUACAAUUUCUGGCUUCtt–3′ and 5′–GAGCCGCUGGUGUAUCUUU–3′, respectively. The Silencer Select® scrambled siRNA was purchased from Thermo Fisher Scientific (Waltham, MA) and used as the control. LS‐174 T and HT‐29 cells were transfected with 1 pmol and 5 pmol each of siRNA in a 96‐well and 24‐well culture dish (4 × 10^5^ cells/ml) using the Lipofectamine® RNAiMAX Transfection Reagent (Invitrogen), as previously described.^12^, ^13^, ^14^, ^15^, ^17^, ^18^

### 2.9 Data analysis

Results are expressed as mean ± SE. Statistical significance was determined by Student's *t*‐test using the graphpad prism software (graphpad Software).

## 3 RESULTS

### 3.1 LPS induced *REG IV* mRNA

**FIGURE 1:**
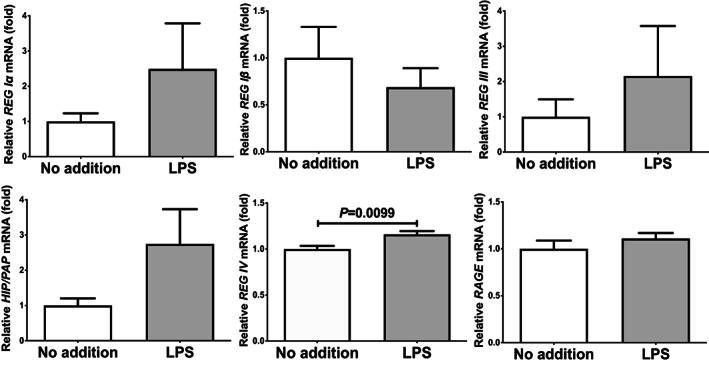
mRNA levels of *REG* family genes (*REG Iα*, *REG Iβ*, *REG III*, *HIP/PAP* and *REG IV*) and *RAGE* in LS‐174 T human intestinal epithelial cells stimulated by LPS (1 μg/ml) for 24 h. The mRNAs were measured by real‐time RT‐PCR using *β‐actin* as an endogenous control. Data are expressed as mean ± SE for each group (*n* = 4). The statistical analyses were performed using Student's *t*‐test

**FIGURE 2:**
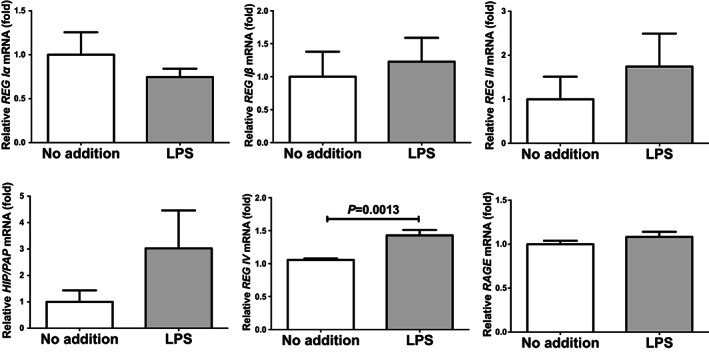
mRNA levels of *REG* family genes (*REG Iα*, *REG Iβ*, *REG III*, *HIP/PAP* and *REG IV*) and *RAGE* in HT‐29 human intestinal epithelial cells stimulated by LPS (1 μg/ml) for 24 h. The mRNAs were measured by real‐time RT‐PCR using *β‐actin* as an endogenous control. Data are expressed as mean ± SE for each group (*n* = 4). The statistical analyses were performed using Student's *t*‐test

We added 1 μg/ml LPS in the culture medium of LS‐174 T and HT‐29 human intestinal epithelial cells and cultured for 24 h. After the stimulation, we prepared RNA from the cells and REG IV expression was measured by real‐time RT‐PCR. As shown in Figures 1 and 2, *REG IV* mRNA was significantly increased in response to the LPS addition in both LS‐174 and HT‐29 human intestinal epithelial cells but not increased mRNAs of the other members of human *REG* family (*REG Iα*, *REG Iβ*, *REG III* and *HIP/PAP*) and *RAGE*.

### 3.2 REG IV acts as a growth factor in human intestinal epithelial cells

**FIGURE 3:**
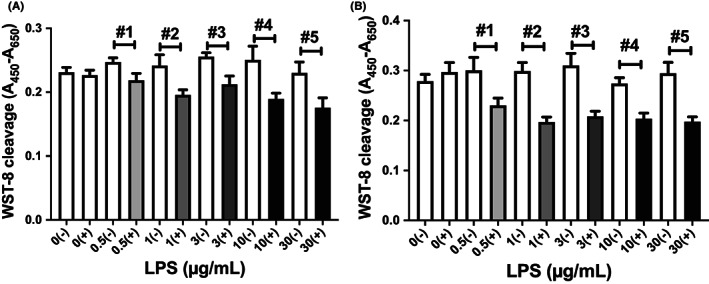
Effects of siRNA transfection on cell proliferation in (A) LS‐174 T and (B) HT‐29 human intestinal epithelial cells stimulated by LPS. After transfection of siRNA against *REG IV* into LS‐174 T and HT‐29 cells, LPS (0–30 μg/ml) was added. Cellular proliferation was measured by WST‐8 assay. Data are expressed as mean ± SE for each group (*n* = 4–8). 0, 0.5, 1, 3, 10 and 30 mean LPS concentrations (μg/ml). (−): Scrambled RNA, (+): siREG IV RNA, #1: *p* = 0.0490, #2: *p* = 0.0267, #3: *p* = 0.0288, #4: *p* = 0.0212, #5: *p* = 0.0325 in LS‐174 T cells (A). #1: *p* = 0.0342, #2: *p* = 0.0002, #3: *p* = 0.0018, #4: *p* = 0.0008, #5: *p* = 0.0013 in HT‐29 cells (B). The statistical analyses were performed using Student's *t*‐test

In order to confirm REG IV acts a growth factor in intestinal epithelial cells, we introduced siREG IV RNA into LS‐174 and HT‐29 cells and added LPS (0–30 μg/ml) into the medium. As shown in Figure 3, cell proliferation was significantly inhibited by LPS (0.5–30 μg/ml) in siRAGE IV‐introduced cells (‘+’ in Figure 3). In contrast, inhibitory effects on cell proliferation by LPS (0.5–30 μg/ml) were not shown in scrambled RNA introduced cells (‘−’ in Figure 3), suggesting that REG IV expression in intestinal epithelial cells in response to LPS stimulation and that REG IV works as a growth factor for intestinal epithelial cells in the presence of LPS.

### 3.3 The promoter activities of *REG IV* were not increased by LPS

**FIGURE 4:**
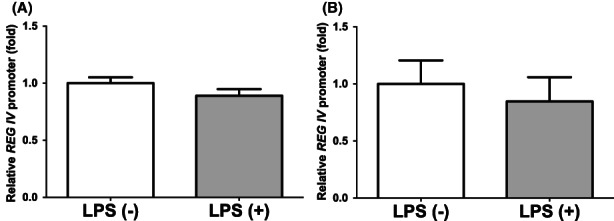
Luciferase reporter assays of promoter activities of *REG IV* in (A) LS‐174 T and (B) HT‐29 human intestinal epithelial cells. Reporter plasmid prepared by inserting the promoter fragments of *REG IV* (−1053 ~ +22) upstream of a firefly luciferase reporter gene in pGL3‐Basic vector was transfected into LS‐174 T and HT‐29 human intestinal epithelial cells. After cells were stimulated by LPS (1 μg/ml) for 24 h, the cells were lysed, and the promoter activities of *REG IV* were measured using β‐galactosidase as a transfection efficiency control. All data are represented as the mean ± SE of the samples (*n* = 4). The statistical analyses were performed using Student's *t*‐test

In order to determine whether the LPS‐induced increases in *REG IV* mRNAs were caused by activation of transcription of *REG IV* gene, a 1075 bp fragment containing 1053 bp of the *REG IV* promoter was fused to the luciferase gene of pGL3‐Basic vector and transfected it into LS‐174 T and HT‐29 human intestinal epithelial cells. After LPS stimulation, we measured promoter activities and found that *REG IV* promoter activity was not significantly increased by LPS in LS‐174 T nor HT‐29 human intestinal epithelial cells (Figure 4). These results strongly suggested that the gene expression of *REG IV* in response to LPS stimulation was not regulated by transcription.

### 3.4 LPS reduced microRNA‐24 in human intestinal epithelial cells

**FIGURE 5:**
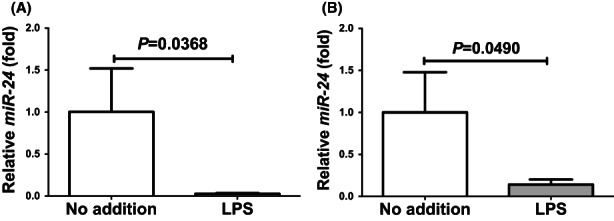
MicroRNA‐24 levels in (A) LS‐174 T and (B) HT‐29 human intestinal epithelial cells stimulated by LPS (1 μg/ml) for 24 h. The miRNAs were measured by real‐time RT‐PCR using U6 RNA as an endogenous control. Data are expressed as mean ± SE for each group (*n* = 4). The statistical analyses were performed using Student's *t*‐test

We considered a possible explanation that the LPS‐induced upregulation of REG IV (Figures 1 and 2) was controlled post‐transcriptionally. Therefore, we searched targeted miRNA (miR) using the MicroRNA.org program (http://www.microrna.org/microrna/home.do), which revealed that *REG IV* mRNA has a potential target sequence for miR‐24. We then measured the miR‐24 levels of LPS‐treated cells by real‐time RT‐PCR and found that the miR‐24 levels in LPS‐treated cells were significantly lower than that of untreated cells (Figure 5: 0.02782 ± 0.006653 fold, *p* = 0.0368 in LS‐174 T cells; 0.1400 ± 0.06179 fold, *p* = 0.0490 in HT‐29 cells).

### 3.5 *REG IV* mRNA levels were regulated by microRNA‐24 in human intestinal cells

**FIGURE 6:**
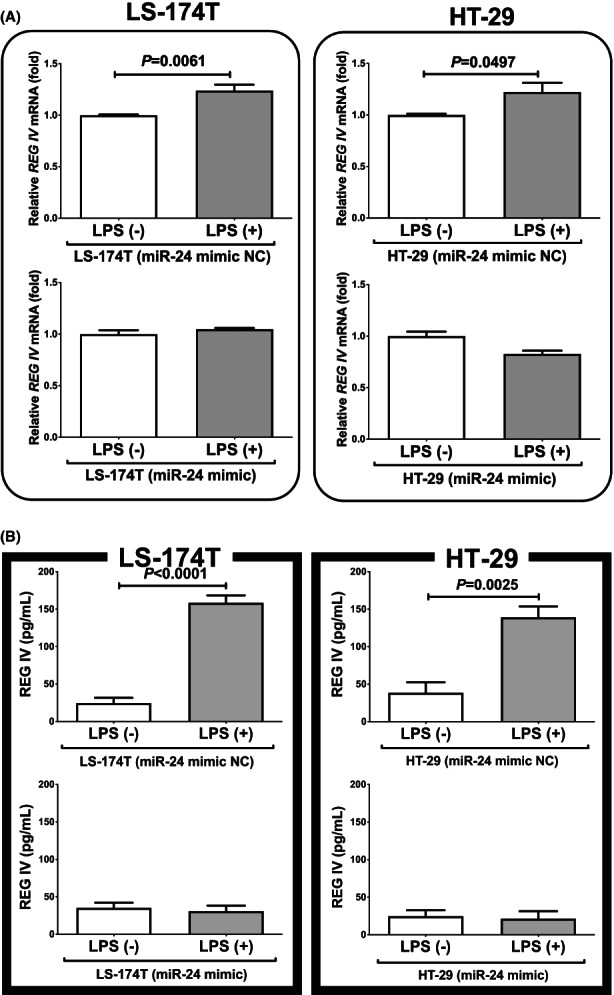
Effects of miR‐24 mimic transfection on *REG IV* expression. The miR‐24 mimic (5’–UGGCUCAGUUCAGCAGGAACAGtt–3′ and 5’–CUGUUCCUGCUGAACUGAGCCAtt–3′) and non‐specific control RNA (miR‐24 mimic NC) (5’–UUCUCCGAACGUGUCACGUtt–3′ and 5’–ACGUGACACGUUCGGAGAAtt–3′) were synthesized by NGRL and introduced into LS‐174 T and HT‐29 human intestinal epithelial cells using Lipofectamine® RNAiMAX just before LPS addition, and the mRNA levels of *REG IV* (A) were measured by real‐time RT‐PCR using *β‐actin* as an endogenous control. The levels of REG IV in the cell culture medium (B) were measured by elisa. Data were expressed as mean ± SE for each group (*n* = 4). The statistical analyses were performed using Student's *t*‐test

In order to investigate whether *REG IV* expression in LPS‐treated cells is regulated by miR‐24, miR‐24 mimic and non‐specific control RNA (miR‐24 mimic NC) were introduced into LS‐174 T and HT‐29 human intestinal epithelial cells with LPS exposure, and the mRNA levels of *REG IV* and REG IV protein levels in cell culture medium were measured by real‐time RT‐PCR and elisa, respectively. As shown in Figure 6, we found that the LPS‐induced increases in *REG IV* mRNA and REG IV protein in culture medium were abolished by the introduction of miR‐24 mimic but not by miR‐24 mimic NC. These findings indicate that LPS down‐regulates the miR‐24 level in human intestinal epithelial cells (Figure 5) and that the levels of *REG IV* mRNA and REG IV protein are increased via the miR‐24 mediated mechanism.

### 3.6 LPS induced REG IV expression via RAGE and TLR4

**FIGURE 7:**
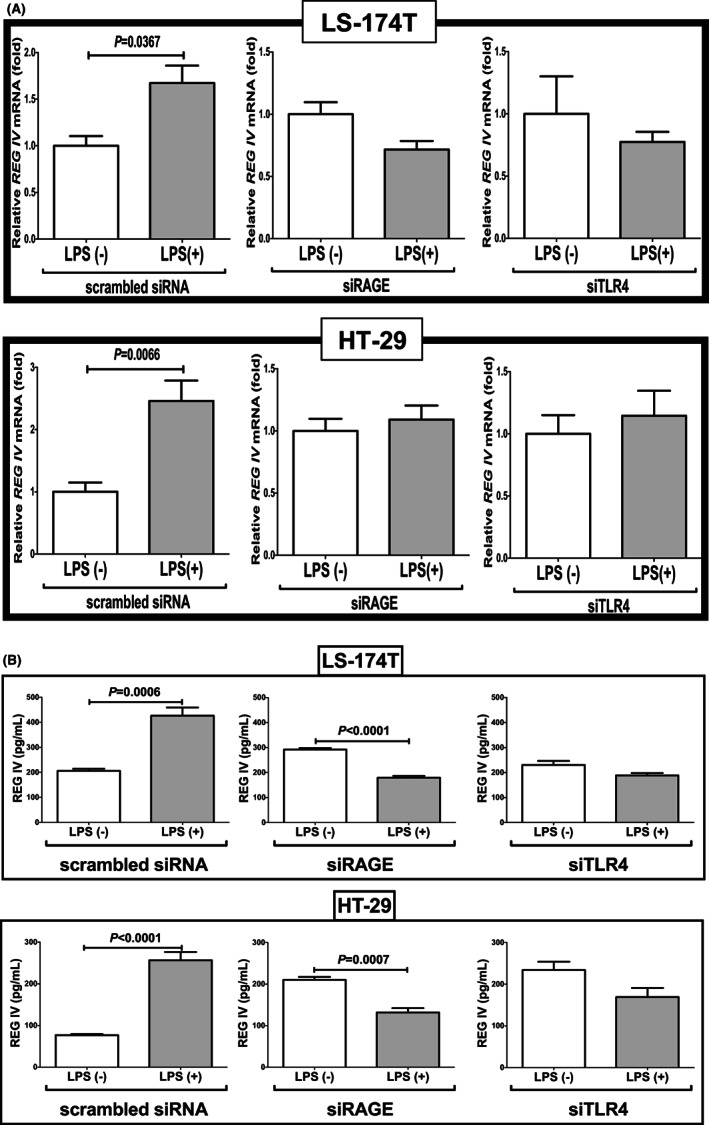
Effects of siRNAs directed against *RAGE* and *TLR4* on the LPS‐induced gene expression of REG IV. After introduction of siRNAs for *RAGE* and *TLR4* into LS‐174 T and HT‐29 human intestinal epithelial cells, the cells were incubated with 1 μg/ml LPS for 24 h. (A) The levels of *REG IV* mRNA were measured via real‐time RT‐PCR using *β‐actin* as an endogenous control. (B) The levels of REG IV in the cell culture medium were measured via elisa. Data are expressed as mean ± SE for each group (*n* = 4). The statistical analyses were performed using Student's *t*‐test

LPS has been reported to act as a RAGE ligand^20^ and RAGE ligands, such as AGE and HMGB1 induced *REG IV* gene expression in human LS‐174 T cells (data not shown). It is well known that RAGE was upregulated in some cells such as adipocytes by RAGE ligands such as AGE and HMGB1^15^ and we found significant upregulation of *RAGE* mRNA in response to AGE and HMGB1 in LS‐174 T cells (data not shown). As RAGE and TLR4 are considered as receptors for LPS to transduce LPS signal into cells,^20^, ^21^, ^22^ we investigated which signalling pathway is used in the LPS‐induced REG IV expression in human intestinal epithelial cells using siRNA introductions for RAGE and TLR4. The LPS‐induced upregulations of *REG IV* mRNA and REG IV protein in culture medium were clearly abolished in both LS‐174 T and HT‐29 human intestinal epithelial cells by the introduction of siRNAs both for RAGE and TLR4 (Figure 7).

## 4 DISCUSSION

Although overexpression of REG IV has been reported in IBD,^11^, ^23^, ^24^, ^25^ the mechanisms of *REG IV* induction have been elusive.^12^, ^13^ In cancer cells, overexpression of *REG IV* gene was also reported in colorectal carcinoma,^26^, ^27^, ^28^, ^29^, ^30^ gastric cancer,^31^, ^32^, ^33^, ^34^ gallbladder carcinoma,^35^, ^36^ prostate cancer,^37^, ^38^ pancreatic cancer,^39^, ^40^, ^41^ glioma^42^ and ovarian carcinoma.^43^, ^44^, ^45^ Despite lots of reports described above, the mechanisms of REG IV induction in cancer cells and in inflammatory cells/tissues have also been elusive.

In this study, we demonstrated that LPS induced increases of REG IV mRNA and protein in human intestinal epithelial cells. We further studied the mechanisms by which LPS upregulates the gene expression of *REG IV* and found that the LPS‐signal was transduced via RAGE and TLR4 receptors and that the *REG IV* mRNA level in human intestinal epithelial cells was post‐transcriptionally regulated via miR‐24‐regulated mechanism.

Originally, *Reg* was isolated as a gene specifically expressed in regenerating rat islets induced by 90% pancreatectomy with PARP inhibitor administrations.^2^, ^3^ The *Re*g and *Reg*‐related genes were isolated and were revealed to constitute a multigene family, the *Reg* gene family. Based on the primary structures of the Reg proteins, the members of the family are grouped into four subclasses: types I, II, III and IV.^1^ In humans, five *REG* family genes, that is *REG Iα*, *REG Iβ*, *RS*, *HIP/PAP* and *REG III* are tandemly ordered on chromosome 2p12, while *REG IV* locates on chromosome 1.^1^ In the mouse genome, all *Reg* family genes except for *Reg IV* (i.e. *Reg I*, *Reg II*, *Reg IIIα*, *Reg IIIβ*, *Reg IIIγ* and *Reg IIIδ*) were mapped to a contiguous 75 kbp region of chromosome 6C,^46^ while *Reg IV* was mapped on chromosome 3.^47^ Type I (and type II) Reg proteins are expressed in regenerating islets and are involved in β‐cell regeneration.^1^, ^3^, ^4^, ^19^, ^48^ Reg family proteins have been suggested to be involved in cellular proliferation in gastrointestinal cells, hepatic cells, cardiovascular cells and neuronal cells.^1^ Reg protein was also shown to mediate gastrointestinal epithelial cell proliferation in rats.^6^, ^49^ Yonemura et al.^8^ showed that the expression of the *REG Iα* gene is closely related to the infiltrating property of gastric carcinoma and may be a prognostic indicator of differentiated adenocarcinomas of the stomach. These observations suggest that the *Reg* gene family is involved in cell proliferation in a variety of cell types, including gastrointestinal cells. Concerning human *REG* family gene expression in IBD, upregulations of *REG Iα*,^12^, ^13^, ^24^, ^50^ *REG Iβ*,^12^, ^13^, ^24^ *REG III*,^10^ *HIP/PAP* ^10^, ^24^, ^51^ and *REG IV* ^11^, ^12^, ^13^, ^24^ were reported. The mechanisms of upregulation in *REG Iα* and *REG Iβ* in IBD were clarified,^12^ and some upregulation mechanisms of mouse *Reg III* genes^52^ and human *REG IIIα* (*HIP/PAP*) gene^53^ were also reported.

Upregulation of *REG IV* gene in IBD has been reported previously.^11^, ^23^, ^24^, ^25^ However, the mechanism of *REG IV* expression in the colon is still controversial: caudal‐type homeobox transcription factor 2 (CDX2) and GATA DNA‐binding protein 6 (GATA6) involvements were reported. In our previous study, we found that *REG IV* mRNA was significantly upregulated in UC and CD samples and downregulated by the addition of TNFα in cultured colon cells.^12^ We therefore introduced siRNAs for *CDX2*, *GATA6* and suppressors of the cytokine signalling 3 (SOCS3) into LS‐174 T human intestinal epithelial cells and evaluated the effect of the siRNAs on the TNFα‐induced suppression of *REG IV*. The introduction of *GATA6* siRNA, but not *CDX2* or *SOCS3* siRNA, significantly attenuated the TNFα‐induced *REG IV* suppression, indicating that GATA6 is essential for *REG IV* expression and TNFα‐induced *REG IV* suppression in colon epithelial cells.^13^ Recently, miRs were reported to regulate some mRNA levels via degradation of target mRNAs.^54^ MiR‐24 was reported to reduce *REG IV* mRNA,^55^ and miR‐143 and miR‐363 were reported to inhibit cell proliferation via downregulation of *GATA6* mRNA.^56^, ^57^ We found that miR‐363, but not miR‐24 or ‐143, was significantly increased by TNFα stimulation and that the introduction of miR‐363 mimic abolished the TNFα‐induced *REG IV* downregulation.^13^

Although REG IV has been reported as a novel target for the enrichment of intestinal epithelial stem cells and mucosal healing,^58^ the mechanism of upregulation of REG IV in human intestinal biopsies from IBD patients has still been unrevealed. The main reasons why the mechanism of REG IV upregulation in human intestinal epithelial cells has been unrevealed may be that we have been unable to establish an in vitro model of REG IV induction in human intestinal epithelial cells. In this aspect, *Reg IV* expression was not analysed in bacterial reconstitution model of mouse colitis^10^ although the model would be a good model to see Reg IV expression in colitis. Sun et al.^59^ reported that *Reg IV* expression was predominant from the duodenum to the distal colon and that the expression level peaked in the colon in untreated and indomethacin‐induced colitis mice. In the present study, we first found that LPS induced REG IV expression in human intestinal epithelial cells in vitro. The findings enable us to investigate how REG IV upregulation occurs not only in the LPS‐stimulated cells but also LPS‐stimulated animal models.

In this study, we showed that REG IV expression was induced by LPS in human intestinal epithelial cells via downregulation of miR‐24. The LPS signal was mediated by RAGE/TLR4 receptors in intestinal epithelial cells. In IBD conditions, damaged intestinal cells were replenished by proliferation of intestinal cells. REG IV could work to proliferate intestinal cells to replenish intestinal mucosa as it is a growth factor for intestinal epithelial cells.

## AUTHOR CONTRIBUTIONS

**Shin Takasawa:** Conceptualization (lead); data curation (equal); formal analysis (equal); methodology (equal); writing – original draft (lead); writing – review and editing (equal). **Chikatsugu Tsuchida:** Methodology (equal); writing – review and editing (equal). **Sumiyo Sakuramoto‐Tsuchida:** Investigation (equal); methodology (equal); writing – review and editing (equal). **Tomoko Uchiyama:** Investigation (equal); methodology (equal); writing – review and editing (equal). **Mai Makino:** Investigation (equal); methodology (equal); writing – review and editing (equal). **Akiyo Yamauchi:** Investigation (equal); methodology (equal); writing – review and editing (equal). **Asako Itaya‐Hironaka:** Data curation (equal); formal analysis (equal); investigation (equal); methodology (equal); writing – review and editing (equal).

## CONFLICT OF INTEREST

The authors confirm that there are no conflicts of interest.

## Data Availability

The data that support the findings of this study are available from the corresponding author upon reasonable request.
